# Engaging in physical activity instead of (over)using the smartphone: An experimental investigation of lifestyle interventions to prevent problematic smartphone use and to promote mental health

**DOI:** 10.1007/s10389-023-01832-5

**Published:** 2023-02-09

**Authors:** Lena-Marie Precht, Franziska Mertens, Debora S. Brickau, Romy J. Kramm, Jürgen Margraf, Jan Stirnberg, Julia Brailovskaia

**Affiliations:** Mental Health Research and Treatment Center, Department of Clinical Psychology and Psychotherapy, Ruhr-Universität Bochum, Massenbergstr. 9-13, 44787 Bochum, Germany

**Keywords:** Problematic smartphone use, Reduction of smartphone use, Increase in physical activity, Mental health, Lifestyle interventions

## Abstract

**Aim:**

Tendencies of problematic smartphone use (PSU) have risen during the past decade. As PSU is consistently linked to mental health issues, measures to prevent its appearance and to promote mental health are urgently required.

**Subject and Methods:**

The present study investigated the impact of three interventions on health behavior, PSU, positive mental health (PMH), and depression and anxiety symptoms. Overall, 503 persons from Germany (*M*_age_ = 29.19, *SD*_age_ = 10.51, range: 18–79) participated in the study. Over 14 days, the three experimental groups (a) reduced their daily smartphone use time by 60 minutes, (b) increased their daily level of physical activity by 30 minutes, and (c) combined both measures. The control group continued its behavior as usual. Outcomes were assessed via online surveys at five measurement time points (baseline, intermediate, post-intervention, and 1 and 3 months after the intervention).

**Results:**

All interventions resulted in a significant increase in weekly physical activity and in reduced symptoms of PSU, depression, and anxiety. Furthermore, the smartphone reduction and the combination of both measures contributed to a significant reduction of participants’ daily smartphone use and higher levels of PMH. The effects of the reduction of smartphone use time and its combination with increased physical activity were more stable in the longer term than the increase in physical activity only.

**Conclusion:**

Combined with an increase in physical activity, the reduction of smartphone use time could serve as an efficient and cost-effective measure for the prevention of PSU and the promotion of mental health.

## Introduction

Smartphones are ubiquitous nowadays. Apart from the continuing growth in worldwide smartphone ownership (O'Dea 2022), the time spent using smartphones is also rising (Harvey et al. 2022). In 2019, the average time spent on the device accounted for around 3 hours per day (e.g., MacKay 2019; Wurmser 2019). However, recent studies indicated a further increase in screen time as a consequence of the COVID-19 pandemic (Jiang et al. 2022; Saadeh et al. 2021). Because of their numerous functions and applications as well as the possibility for quick and permanent access to the Internet, smartphones have a significant impact on everyday life. They help us in organizing daily routines, enable an almost endless supply of information, and offer a wide range of entertainment. Furthermore, they provide the opportunity to stay in contact with family and friends and, in particular by means of social media, to connect with new people (Radtke et al. 2022; Ting and Chen 2020). However, despite the many benefits, smartphone use can result in negative outcomes when use becomes excessive and when a person loses control over their smartphone use.

Notably, not everyone who frequently engages in smartphone use develops addictive use tendencies. Recent research provides a theoretical framework for the explanation of this phenomenon. The Interaction of Person-Affect-Cognition-Execution (I-PACE) model (Brand et al. 2016, 2019) describes various psychological and neurobiological processes which can result in the development and persistence of specific Internet use disorders. According to this model, the interaction of specific personal characteristics (e.g., genetics, personality, or psychopathology) with certain moderators and mediators (e.g., coping style or Internet-related cognitive biases) can lead to positive reinforcement of smartphone use. However, in the longer term this can lead to alterations in affective and cognitive responses to specific device-related stimuli as well as executive functions (e.g., inhibitory control and decision-making). For example, when people lack functional coping strategies and use the smartphone to escape stress and negative emotions, they might feel relief in the short-term, which reinforces the reliance on the smartphone in similar situations in the future (Brand et al. 2016). However, in the longer term, the habitual smartphone use replaces more adaptive coping strategies and thus prevents an appropriate solution of their problems (Ting and Chen 2020).

Although numerous studies address the topic of smartphone addiction, the evidence supporting excessive use as a behavioral addiction is scarce and critically discussed (e.g., Billieux et al. 2015; Yu and Sussman 2020). Addictive smartphone use is not yet considered a formal clinical disorder in diagnostic manuals (American Psychiatric Association 2013; World Health Organization 2019). Therefore, we follow the recommendation of Panova and Carbonell (2018) and use the term “problematic smartphone use” (PSU) in the present study. PSU is conceptualized as a compulsive pattern of smartphone use that impairs daily functioning (Busch and McCarthy 2021) and shares similar characteristics with other addictive behaviors such as cognitive salience, mood modification, tolerance, withdrawal, conflict, or relapse (Billieux et al. 2015; Griffiths 2005).

An extensive body of research describes associations between frequent smartphone use as well as PSU and several detrimental outcomes (Thomée 2018). In addition to physical health issues (Reer et al. 2022; Ting and Chen 2020), neurological changes (Ratan et al. 2021), and the occurrence of accidents (Kim et al. 2017), previous studies also reported relations with lower work productivity (Duke and Montag 2017) and academic achievement (Sunday et al. 2021). Mental health issues are the most frequently identified consequences of PSU (Busch and McCarthy 2021). As reviewed by Elhai et al. (2017), PSU is significantly positively related to depression and anxiety severity. Furthermore, it is positively associated with impairments in sleep, such as shorter sleep and poor sleep quality (Thomée 2018; Yang et al. 2020). In addition, studies have demonstrated positive correlations between smartphone use and symptoms of stress (Vahedi and Saiphoo 2018), PSU, and loneliness (Lapierre et al. 2019; Thomée 2018).

Taken together, results suggest that smartphones can lead to several adverse outcomes when they are overused. This is of particular importance due to an increase in PSU that has been noted in the past decade (Olson et al. 2020) and that has been further intensified in the course of the COVID-19 pandemic (Masaeli and Farhadi 2021). However, the majority of the existing studies regarding this issue used cross-sectional designs and therefore did not allow valid conclusions on causal directions of the associations (Thomée 2018). It seems reasonable that the relationships are complex, and the different constructs may be related in a circular manner (Elhai et al. 2017; Stanković et al. 2021). Consequently, longitudinal experimental designs for true causal conclusions regarding the effects of smartphone use are needed. Furthermore, it is essential to identify and test simple measures that are most likely to bring PSU back to healthy levels or alternatively to prevent its development (Busch and McCarthy 2021).

“Digital detox” (i.e., refraining from electronic devices like the smartphone for certain periods of time) has been suggested as a possibility for reducing the negative impact of smartphone use on different health-related outcomes (Radtke et al. 2022). The first experimental studies addressing digital detox targeted social media use. Tromholt (2016) asked 516 participants to quit Facebook use for 1 week and found positive effects of the abstinence on life satisfaction and affective well-being. Hunt et al. (2018) found evidence for reduced levels of depression and loneliness after limiting the use of social media platforms via mobile phone by 30 minutes per day over a period of 3 weeks. Another research group examined the effects on mental health of a 20-minute reduction in daily Facebook use for a period of 2 weeks (Brailovskaia et al. 2020b). In addition to a significant increase in life satisfaction, the intervention led to a significant decrease in problematic Facebook use and depressive symptoms. In summary, results of the studies indicate that both complete abstinence and reduction in social media use can result in positive health outcomes.

Recent studies explored whether the positive effects of social media detox also apply for smartphone use. One-week abstinence from smartphone use in the bedroom led to higher levels of subjective happiness and quality of life as well as reduced levels of PSU (Hughes and Burke 2018). In another study, participants were asked to try to reduce their smartphone use, i.e., there was no specified amount of reduction desired, for 2 weeks. Results indicated improvements in symptoms of depression, perceived stress, and sleep quality in persons with moderate depression symptoms at baseline (Liao 2019). However, neither study investigated the long-term effects of the respective interventions. Furthermore, they only investigated the effects of smartphone use reduction and not complete abstinence from smartphone use. Brailovskaia et al. (2022) addressed both limitations by investigating short- and long-term effects (up to 4 months after the intervention) of two experimental conditions: the first group abstained completely from smartphone use for 1 week, while the second group reduced its use by 1 hour per day over the same period. Both interventions led to reductions in daily smartphone use and PSU as well as symptoms of anxiety and depression. Further, both groups revealed an increase in life satisfaction (Brailovskaia et al. 2022). In accord with the results of the Facebook reduction study of Brailovskaia et al. (2020b), the frequency of physical activity increased, although it had not been specifically addressed by the experimental manipulation. Most of the effects were stronger and more stable over the 4 months in the reduction group than in the abstinence group. Therefore, the authors concluded that complete abstinence from smartphone use is not necessary for positive effects on different health parameters (Brailovskaia et al. 2022).

Aside from digital detox, engagement in physical activity can also serve as an effective measure for reducing PSU and its detrimental health outcomes. Physical activity is generally accepted as having positive effects on mental health and well-being (Mikkelsen et al. 2017; Wiese et al. 2018). Its positive impact on symptoms of depression and anxiety has been proven in both clinical (Ashdown-Franks et al. 2020) and nonclinical populations (Rebar et al. 2015). Regular moderate physical activity was also shown to have beneficial effects on sleep quality (Kredlow et al. 2015; Wang and Boros 2021). In terms of PSU, physical inactivity was linked to increased smartphone use (Grimaldi-Puyana et al. 2020) as well as higher levels of PSU in high school (Pereira et al. 2020) and college students (Haripriya et al. 2019; Yang et al. 2021). Furthermore, problematic smartphone users engaged in less physical activity (Kim et al. 2015) and were more likely to report forgoing physical activity in order to engage in sedentary behavior with their smartphones (Lepp et al. 2013). Consequently, the studies indicated the effects of physical activity on smartphone use and vice versa. However, due to the lack of longitudinal designs, final conclusions regarding the direction of effects remain unclear.

Against this background, the present study aimed to provide simple measures for the prevention of PSU and detrimental health outcomes, and for the promotion of psychological well-being. Therefore, we investigated the effects of a controlled experimental (a) reduction in smartphone use time, (b) increase in physical activity, and (c) a combination of both measures on longer term smartphone use and physical activity as well as on PSU and mental health. Previous research suggested past behavior to predict current behavior (Nudelman et al. 2021; Ouellette and Wood 1998). In addition, a recent study reported long-term effects of a short-term smartphone reduction on smartphone use time (Brailovskaia et al. 2022). Therefore, we hypothesized that a controlled reduction of smartphone use time would result in reduced smartphone use time not only for the period of the experimental manipulation but also in the longer term (i.e., over the investigated period of 3 months) (Hypothesis 1a). Further, as physical activity was shown to be predictive of long-term exercise habits (Fleig et al. 2014), we also expected higher physical activity levels in the short and longer term as a consequence of the controlled increase in physical activity (Hypothesis 1b). Since many health behaviors (i.e., actions taken by individuals that can enhance or degrade health outcomes; Short and Mollborn 2015) are interrelated and can influence each other (Nudelman et al. 2019; Tan et al. 2018), we further hypothesized an interplay between smartphone use and physical activity: the reduction in smartphone use time would lead to an increase in physical activity (Hypothesis 1c), and the increased physical activity would be followed by a reduction in smartphone use time (Hypothesis 1d).

As it has been shown that higher levels of smartphone use can lead to PSU (Lapierre et al. 2019), we hypothesized that a controlled reduction of smartphone use time would lead to lower levels of PSU (Hypothesis 2a). Further, earlier research suggested that physical activity can support people in satisfying basic psychological needs like the experience of competence or relatedness (Ryan and Deci 2000; Wiese et al. 2018). Thus, engaging in physical activity could reduce the tendency to rely on smartphone use to satisfy one’s needs (Cheng and Lau 2022; Schneider et al. 2022). Therefore, we hypothesized that an increase in physical activity would also reduce levels of PSU (Hypothesis 2b).

Since mental health is more than the absence of mental disorders (as described by two-factor models; e.g., Keyes 2005; Ryff et al. 2006), it is important to distinguish positive (i.e., psychological well-being) and negative aspects (i.e., psychopathology) of mental health (Meier and Reinecke 2021; Schneider et al. 2022). Therefore, we assumed that both interventions would enhance positive mental health (PMH; i.e., the presence of general emotional, psychological, and social well-being; Lukat et al. 2016) (Hypotheses 3a and 3b) and reduce symptoms of depression (Hypotheses 3c and 3d) and anxiety (Hypotheses 3e and 3f).

To the best of our knowledge, this is the first study to compare the effects of a controlled experimental reduction in smartphone use time and an increase in physical activity on PSU and mental health. Moreover, the reciprocal influence of both health behaviors is considered. Furthermore, we also wanted to investigate whether a combination of smartphone use reduction and simultaneous increase in physical activity could be an even more beneficial measure. Following Brailovskaia et al. (2022), we formulated research questions to avoid speculation when comparing the three interventions: *Research Question 1*: Do the effects of the interventions on PSU, mental health, and health behaviors differ? *Research Question 2*: If the effects differ, how do they differ?

## Material and methods

### Procedure

The present study had an experimental longitudinal design with three experimental groups (EG1, EG2, EG3) and a control group (CG). The experimental manipulation lasted for 14 days. While participants of EG1 reduced their daily smartphone use, participants of EG2 increased their daily level of physical activity during this period. In EG3, participants reduced their daily smartphone use and increased their daily level of physical activity. The CG continued its smartphone use and physical activity as usual.

According to Brailovskaia et al. (2022), participants of EG1 and EG3 were advised to reduce their daily smartphone use by 60 minutes for 14 consecutive days. The reduction referred to any Internet-based or non-Internet-based applications and functions. To determine the increase in the daily physical activity level, we followed the World Health Organization (WHO) guidelines on physical activity suggesting that adults aged 18 to 64 years should engage in at least 150–300 minutes of moderate-intensity aerobic physical activity throughout the week (World Health Organization 2020). To ensure that participants reached the recommended amount, even if they did not comply with their task over the entire experimental manipulation period, we asked the participants of EG2 and EG3 to increase their daily level of physical activity by 30 minutes. Jogging, cycling, swimming, weight training, and fast walking were given as examples for possible activities.

Data for all groups were collected at five measurement time points via online surveys. Participants received the online links to the German-language surveys by email. Measurements took place 1 day before the 14-day experimental manipulation started (T1; baseline, day 0), in the middle (T2; intermediate, day 7) and at the end (T3; post-intervention, day 15) of the manipulation period, and 1 (T4; follow-up 1) and 3 months (T5; follow-up 2) after the post-intervention measure. The intermediate and post-intervention measurement (T2 and T3) served for the investigation of short-term effects and the follow-up measurements (T4 and T5) for the investigation of the longer term effects of the experimental manipulation.

### Participants

Data collection took place from April 2021 to May 2022. Overall, 503 persons (80.5% women; *M*_age_ = 29.19, *SD*_age_ = 10.51, range: 18–79) participated in the study. Participants were recruited through flyers that were distributed via mailing lists of different German universities and on social media platforms such as Facebook and Twitter. Calls for participation were also made via television, radio broadcasting, and on respective websites. Implementation of the present study was approved by the responsible ethics committee. Participation requirements included age of 18 years, ownership of a smartphone with a minimum of 1 hour of daily use, the ability to engage in physical activity for at least 30 minutes per day, and a maximum of 1 hour of daily physical activity under common circumstances (i.e., prior to the experimental manipulation period). Participation was voluntary and compensated by course credits for students.

The current study was designed as a randomized controlled trial. Interested persons contacted the principal investigators by email. If the requirements for participation were fulfilled, participants were randomly assigned to one of the four groups according to simple randomization. Subsequently, they received an email with the link to the baseline survey. Participants of the three experimental groups also received a “compliance diary” as a Microsoft Word document. The diary served for the daily documentation of whether the participants had complied with the instruction of their experimental condition (0 = *no*, 1 = *yes*). At the beginning of the baseline survey, participants were properly informed about the study and provided informed consent to participate online. The 14-day intervention period started on the day after the baseline survey. After the 2-week period, participants of the experimental groups sent their compliance diaries back to the principal investigators. Compliance to the intervention was recognized when participants fulfilled their task for at least 9 of the 14 days of the intervention (following Brailovskaia et al. 2020b). To prevent increased attention to smartphone use or physical activity, the CG did not receive a compliance diary. Participants of this group only answered the online surveys. The links to the respective surveys were sent out by the principal investigators at the determined time points via email. If participants did not answer the previous survey, they were reminded to complete the survey up to five times before they were excluded from study participation.

#### Experimental group 1 (EG1): Smartphone reduction

Overall, 141 persons were assigned to the smartphone reduction condition. During the investigation period, 13 persons (9.2%) dropped out. Thus, EG1 comprised 128 participants (81.3% women; *M*_age_ = 28.65, *SD*_age_ = 9.71, range: 18–79) who completed all surveys. All participants were Caucasian. Most of them were university students (57.8%) or employees (38.3%). The remaining participants were trainees (1.6%), freelancers (0.8%), or unemployed (1.6%); 46.9% were in a relationship but not married, 34.4% were single, 14.9% were married, and 3.9% were divorced. Analyses of the compliance diaries revealed a compliance rate of 85.2% (*n* = 109).

#### Experimental group 2 (EG2): Physical activity increase

Overall, 145 persons were assigned to the physical activity increase condition. Since 25 persons (17.2%) dropped out, EG2 comprised 120 participants (82.5% women; *M*_age_ = 29.06, *SD*_age_ = 11.20, range: 18–72), who were all Caucasian; 54.2% of them were university students, 40.8% were employees, 4.2% were freelancers, and one person (0.8%) was a trainee. Most of the participants were in a relationship but not married (52.5%), 30.0% were single, 15.8% were married, and two participants (1.6%) were divorced. Analyses of the compliance diaries revealed a compliance rate of 92.5% (*n* = 111).

#### Experimental group 3 (EG3): Smartphone reduction and physical activity increase

Overall, 146 persons were assigned to the combined condition where participants reduced their smartphone use and increased their level of physical activity. The dropout rate was 9.6% (*n* = 14), so that 132 persons (79.5% women; *M*_age_ = 28.55, *SD*_age_ = 9.71, range: 18–68) were part of EG3. Half of the participants (all Caucasian) were university students (50.8%), 42.4% were employees, 3.8% were freelancers, and 1.5% each were trainees or unemployed; 52.3% were in a relationship but not married, 27.3% were single, 19.0% were married, and two persons (1.5%) were divorced. Analyses of the compliance diaries revealed a compliance rate of 82.6% (*n* = 109).

Following Brailovskaia et al. (2022), non-compliers were kept in the samples for the analyses. Therefore, the results should be considered as conservative estimates of the true treatment effects (Tromholt 2016).

#### Control group (CG)

Overall, 140 persons were assigned to the CG, who only answered the surveys. During the investigation period, 17 persons (12.1%) dropped out. Thus, the CG consisted of 123 participants (78.9% women; *M*_age_ = 30.58, *SD*_age_ = 11.40, range: 18–65), who were all Caucasian; 51.2% of them were university students, 43.9% were employees, 3.3% were freelancers, and one participant each (0.8%) was a trainee or unemployed. In addition, 48.0% were in a relationship but not married, 27.6% were single, 22.0% were married, and 2.4% were divorced.

Analyses of variance (ANOVAs) showed that the participants did not differ regarding demographic variables. This applied for the comparison between the four groups as well as for the comparison of participants who dropped out and those who participated in all surveys. A priori power analyses using the G*Power program (version 3.1; Faul et al. 2009) indicated that a total sample size of at least *N* = 180 (*n* = 45 per group) was required for valid results (repeated-measures ANOVAs, within-between factor design; power > 0.80, *α* = 0.05, effect size *f* = 0.10; Mayr et al. 2007).

### Materials

#### Smartphone use time

Participants were asked to indicate their mean daily smartphone use in minutes. The current mean test–retest reliability calculated over all five measurement time points (*r*_mtrr_) accounted for *r*_mtrr_ = 0.60 (EG1), *r*_mtrr_ = 0.59 (EG2), *r*_mtrr_ = 0.69 (EG3), and *r*_mtrr_ = 0.58 (CG).

#### Physical activity time

The weekly amount of physical activity was measured following the German Physical Activity, Exercise, and Sport Questionnaire (Bewegungs- und Sportaktivität Fragebogen, BSA-F; Fuchs et al. 2015). Participants named up to three different sportive activities (e.g., swimming, cycling, or running) which they had performed during the past week. They also specified the frequency and the duration (in minutes) of each activity. The amounts (frequency multiplied by duration) of all activities were summed to obtain the weekly physical activity time in minutes. Previous research showed that the BSA-F is a valid measure of physical activity time (Fuchs et al. 2015). The current *r*_mtrr_ accounted for *r*_mtrr_ = 0.44 (EG1), *r*_mtrr_ = 0.38 (EG2), *r*_mtrr_ = 0.28 (EG3), and *r*_mtrr_ = 0.43 (CG).

#### Problematic smartphone use

A modified version of the Bergen Social Media Addiction Scale (BSMAS; original English version: Andreassen et al. 2016; German version: Brailovskaia et al. 2020a) was used for the assessment of PSU. The BSMAS was modified by replacing the term “social media” by “smartphone” and by changing the reference period of the “past year” to “in general.” The six items (e.g., “How often have you used the smartphone in order to forget about personal problems?”) address the six core addiction elements (i.e., salience, mood modification, tolerance, withdrawal symptoms, conflict, and relapse; Griffiths 2005) and are rated on a five-point Likert-type scale ranging from *very rarely* (1) to *very often* (5). Brailovskaia et al. (2022) reported scale reliability in the range of *α* = 0.81–0.93 for the modified version of the BSMAS. The current reliability accounted for *α*_EG1_ = 0.79–0.84, *α*_EG2_ = 0.79–0.87, *α*_EG3_ = 79–0.87, and *α*_CG_ = 0.85–0.88.

#### Positive mental health

The Positive Mental Health Scale (PMH-scale; Lukat et al. 2016) served as an indicator for the positive dimension of mental health, i.e., psychological well-being. The instrument includes nine items (e.g., “I enjoy my life”) that are rated on a four-point Likert-type scale (0 = *do not agree*, 3 = *agree*). In previous research, the scale reliability of the PMH-scale was *α* = 0.93 (Lukat et al. 2016; Precht et al. 2022). The current reliability accounted for *α*_EG1_ = 0.87–0.91, *α*_EG2_ = 0.89–0.94, *α*_EG3_ = 0.88–0.92, and *α*_CG_ = 0.91–0.92.

#### Depressive and anxiety symptoms

The depression and anxiety subscales of the Depression Anxiety Stress Scale-21 (DASS-21; original English version: Lovibond and Lovibond 1995; German version: Nilges and Essau 2015) were used to assess symptoms of depression and anxiety as indicators of the negative dimension of mental health. Both scales consist of seven items (e.g., depression subscale: “I felt that I had nothing to look forward to”; anxiety subscale: “I felt I was close to panic”) that refer to the past week and are rated on a four-point Likert-type scale ranging from 0 (*did not apply to me at all*) to 3 (*applies to me very much or most of the time*). Nilges and Essau (2015) reported scale reliability of *α*_depression_ = 0.88 and *α*_anxiety_ = 0.76 in a nonclinical German sample. The current depression scale reliability accounted for *α*_EG1_ = 0.85–0.91, *α*_EG2_ = 0.87–0.92, *α*_EG3_ = 0.84–0.89, and *α*_*CG*_ = 0.88–0.92. The current anxiety scale reliability values were *α*_EG1_ = 0.79–0.86, *α*_EG2_ = 0.64–0.80, *α*_EG3_ = 0.74–0.78, and *α*_CG_ = 0.80–0.88.

For all instruments used, higher scores indicated higher levels of the measured construct. All variables were assessed at all five measurement time points in all groups, except for smartphone use time and physical activity. Both were only measured at baseline, post-intervention, and follow-up measurements since an assessment within the period of the experimental manipulation (intermediate measurement) could have influenced the interventions.

### Statistical analyses

Statistical analyses were conducted using SPSS 27 software. Datasets of the participants who answered all five surveys during the investigation period were complete. Thus, there were no missing data. After descriptive analyses, repeated-measures analyses of variance (ANOVAs; within-between factor design) were conducted to investigate short- and longer term effects of the different interventions as well as for the comparison of the four groups. Because of a significant Mauchly’s test, the Greenhouse–Geisser adjustment ($\varepsilon$) was used to correct for violations of sphericity for all variables. Partial eta-squared (η^2^_p_) served as the effect-size measure of main effects (i.e., measurement time point; group condition) and of interaction effects (i.e., measurement time point $\times$ group condition). For post hoc comparisons between groups, Cohen’s *d* was used as effect-size measure. For post hoc comparisons within groups, Cohen’s *d*_Repeated Measures_ (Morris 2008) was considered as effect-size measure. Due to multiple testing, all post hoc comparisons were Bonferroni-corrected (level of significance: *p* < 0.05, two-tailed).

## Results

Descriptive statistics of the investigated variables for each group at the five measurement time points are summarized in Table 1.

**Table 1:** Descriptive statistics of the investigated variables over five measurement time points

	Group	T1	T2	T3	T4	T5
		*M (SD)*	*M (SD)*	*M (SD)*	*M (SD)*	*M (SD)*
Daily smartphone use time (in minutes)	EG1	239.02 (115.67)		174.63 (100.13)	194.64 (98.02)	176.53 (77.18)
	EG2	226.67 (104.58)		215.48 (196.28)	206.10 (119.69)	213.48 (114.49)
	EG3	238.87 (108.46)		185.99 (100.23)	201.79 (106.00)	209.91 (110.05)
	CG	225.76 (77.67)		209.39 (83.54)	214.49 (76.51)	220.07 (79.77)
Weekly physical activity (in minutes)	EG1	170.50 (178.81)		231.74 (311.85)	210.62 (258.31)	218.55 (297.86)
	EG2	143.55 (179.00)		328.88 (220.11)	226.50 (332.96)	252.82 (297.53)
	EG3	136.38 (183.34)		317.08 (177.08)	195.45 (268.88)	206.33 (220.67)
	CG	177.93 (188.01)		178.28 (212.61)	169.28 (204.10)	176.38 (232.78)
PSU	EG1	13.85 (4.71)	13.84 (4.79)	13.44 (4.93)	12.33 (4.73)	11.26 (4.50)
	EG2	12.63 (4.73)	12.03 (4.87)	11.65 (4.83)	11.97 (4.75)	12.27 (5.35)
	EG3	13.92 (4.85)	12.88 (4.45)	12.56 (4.75)	12.39 (4.68)	12.60 (4.95)
	CG	13.07 (5.20)	12.56 (4.93)	12.64 (5.41)	12.86 (5.06)	12.98 (5.29)
PMH	EG1	19.05 (4.67)	19.59 (5.08)	19.62 (5.30)	20.03 (5.03)	20.39 (4.82)
	EG2	19.04 (4.86)	19.42 (5.17)	19.65 (5.51)	19.23 (5.53)	19.11 (5.96)
	EG3	18.61 (4.81)	18.96 (4.79)	19.40 (4.95)	19.61 (4.65)	19.81 (4.45)
	CG	18.67 (5.42)	18.55 (5.01)	18.41 (5.37)	18.59 (5.20)	18.48 (5.02)
Depressive symptoms	EG1	3.98 (3.93)	3.47 (3.87)	3.27 (3.91)	3.17 (4.01)	2.76 (3.19)
	EG2	4.05 (4.18)	3.58 (3.66)	3.23 (3.44)	3.26 (3.69)	3.71 (4.39)
	EG3	4.37 (4.21)	3.59 (3.82)	3.34 (4.01)	3.35 (3.86)	3.17 (3.26)
	CG	4.02 (4.00)	3.98 (4.23)	4.03 (4.27)	4.01 (4.47)	4.24 (4.44)
Anxiety symptoms	EG1	2.86 (3.27)	2.43 (3.24)	2.30 (3.55)	2.05 (2.81)	1.91 (2.64)
	EG2	2.82 (2.89)	1.96 (2.25)	1.87 (2.48)	2.03 (2.51)	2.28 (2.92)
	EG3	3.23 (3.22)	2.43 (2.95)	1.90 (2.57)	2.32 (2.86)	2.63 (2.85)
	CG	2.98 (3.51)	2.85 (3.39)	2.92 (3.85)	2.94 (3.94)	3.22 (4.20)

*M* = mean, *SD* = standard deviation; EG = experimental group, CG = control group; PSU = problematic smartphone use, PMH = positive mental health; EG1 (smartphone reduction): *n* = 128, EG2 (physical activity increase): *n* = 120, EG3 (smartphone reduction and physical activity increase): *n* = 132, CG: *n* = 123; T1 to T5 = measurement time points (baseline, intermediate, post-intervention, 1-month follow-up, 3-month follow-up, respectively)

**Fig. 1:**
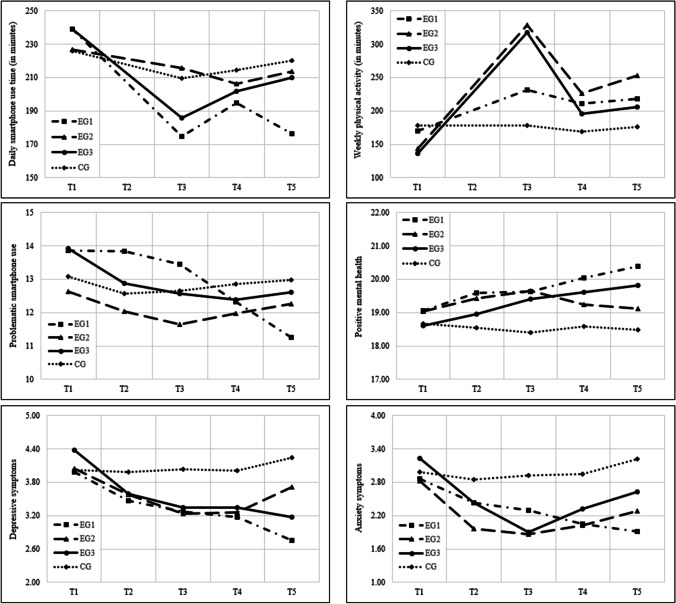
Results of repeated-measures analyses of variance (ANOVAs) for the investigated variables: smartphone use time, physical activity, problematic smartphone use, positive mental health, depressive symptoms, and anxiety symptoms. *Note*. EG = experimental group, CG = control group; EG1 (smartphone reduction): *n* = 128, EG2 (physical activity increase): *n* = 120, EG3 (smartphone reduction and physical activity increase): *n* = 132, CG: *n* = 123; T1 to T5 = measurement time points: baseline, intermediate, post-intervention, 1-month follow-up, 3-month follow-up, respectively

Figure 1 shows the results of the repeated ANOVAs for smartphone use time, physical activity, PSU, PMH, depressive symptoms, and anxiety symptoms.

Pairwise comparisons within the four groups are displayed in Tables 2 and 3, and pairwise comparisons between the groups are shown in Tables 4 and 5.

**Table 2:** Pairwise comparisons of measurement time points within the four groups (T1 to T5; part 1)

	Group	T1 vs. T2		T1 vs. T3		T1 vs. T4		T1 vs. T5		T2 vs. T3	
		md [95%*CI*]	d_RM_	md [95%*CI*]	d_RM_	md [95%*CI*]	d_RM_	md [95%*CI*]	d_RM_	md [95%*CI*]	d_RM_
Daily smartphone use time	EG 1			64.38* [38.52, 90.24]	**.78**	44.38* [23.97, 64.78]	**.42**	62.48* [40.92, 84.05]	**.52**		
	EG 2			11.19 [−15.51, 37.90]		20.57 [−0.51, 41.64]		13.19 [−9.09, 35.47]			
	EG 3			52.88* [27.42, 78.34]	**.74**	37.08* [16.99, 57.18]	**.40**	28.96* [7.72, 50.20]	**.28**		
	CG			16.37 [−10.00, 42.75]		11.28 [−9.54, 32.09]		5.70 [−16.30, 27.70]			
Weekly physical activity	EG 1			−61.24* [−114.13, −8.36]	**.33**	−40.12 [−102.70, 22.46]		−48.05 [−109.74, 13.65]			
	EG 2			−185.33* [−239.95, −130.71]	**.95**	−82.95* [−147.58, −18.32]	**.43**	−109.27* [−172.98, −45.55]	**.51**		
	EG 3			−180.70* [−232.78, −128.62]	**.89**	−59.07 [−120.69, 2.56]		−69.96* [−130.71, −9.20]	**.31**		
	CG			−0.36 [−54.31, 53.59]		8.65 [−55.19, 72.49]		1.55 [−61.39, 64.48]			
PSU	EG 1	0.02 [−0.78, 0.81]		0.41 [−0.49, 1.31]		1.52* [0.59, 2.45]	**.35**	2.59* [1.54, 3.65]	**.50**	0.40 [−0.44, 1.23]	
	EG 2	0.59 [−0.23, 1.41]		0.98* [0.05, 1.90]	**.37**	0.66 [−0.30, 1.62]		0.36 [−0.73, 1.45]		0.38 [−0.48, 1.25]	
	EG 3	1.05* [0.27, 1.82]	**.27**	1.36* [0.48, 2.25]	**.37**	1.54* [0.62, 2.45]	**.43**	1.33* [0.29, 2.36]	**.33**	0.32 [−0.50, 1.14]	
	CG	0.50 [−0.30, 1.31]		0.42 [−0.50, 1.34]		0.20 [−0.74, 1.15]		0.08 [−0.99, 1.16]		−0.08 [−0.93, 0.77]	
PMH	EG 1	−0.54 [−1.29, 0.21]		−0.56 [−1.37, 0.24]		−0.98* [−1.93, −0.03]	**.27**	−1.34* [−2.36, −0.31]	**.36**	−0.02 [−0.71, 0.66]	
	EG 2	−0.38 [−1.15, 0.40]		−0.61 [−1.44, 0.22]		−0.19 [−1.17, 0.79]		−0.07 [−1.12, 0.99]		−0.23 [−0.94, 0.47]	
	EG 3	−0.35 [−1.09, 0.39]		−0.79 [−1.58, 0.01]		−1.00* [−1.94, −0.06]	**.25**	−1.20* [−2.21, −0.19]	**.24**	−0.44 [−1.11, 0.23]	
	CG	0.12 [−0.64, 0.89]		0.27 [−0.55, 1.09]		0.09 [−0.88, 1.06]		0.20 [−0.85, 1.24]		0.15 [−0.55, 0.84]	
Depressive symptoms	EG 1	0.52 [−0.23, 1.26]		0.72 [−0.05, 1.49]		0.81 [−0.06, 1.69]		1.23* [0.30, 2.15]	**.36**	0.20 [−0.46, 0.86]	
	EG 2	0.48 [−0.30, 1.25]		0.83* [0.03, 1.62]	**.26**	0.79 [−0.11, 1.69]		0.34 [−0.61, 1.30]		0.35 [−0.33, 1.03]	
	EG 3	0.78* [0.05, 1.52]	**.25**	1.03* [0.27, 1.79]	**.33**	1.02* [0.16, 1.88]	**.30**	1.20* [0.29, 2.11]	**.31**	0.25 [−0.40, 0.90]	
	CG	0.05 [−0.71, 0.81]		−0.01 [−0.80, 0.78]		0.02 [−0.88, 0.91]		−0.21 [−1.15, 0.73]		−0.06 [−0.73, 0.62]	
Anxiety symptoms	EG 1	0.43 [−0.19, 1.05]		0.56 [−0.10, 1.21]		0.81* [0.14, 1.49]	**.33**	0.95* [0.17, 1.74]	**.34**	0.13 [−0.44, 0.69]	
	EG 2	0.86* [0.22, 1.50]	**.40**	0.95* [0.28, 1.62]	**.42**	0.79* [0.10, 1.49]	**.31**	0.53 [−0.28, 1.35]		0.09 [−0.49, 0.67]	
	EG 3	0.80* [0.19, 1.42]	**.31**	1.33* [0.69, 1.97]	**.51**	0.92* [0.25, 1.58]	**.29**	0.61 [−0.17, 1.38]		0.53 [−0.03, 1.09]	
	CG	0.14 [−0.50, 0.77]		0.07 [−0.60, 0.73]		0.05 [−0.64, 0.74]		−0.24 [−1.04, 0.57]		−0.07 [−0.65, 0.50]	

EG = experimental group, CG = control group; PSU = problematic smartphone use, PMH = positive mental health; EG1 (smartphone reduction): *n* = 128, EG2 (physical activity increase): *n* = 120, EG3 (smartphone reduction and physical activity increase): *n* = 132, CG: *n* = 123; T1 to T5 = measurement time points; md = mean difference, *CI* = confidence interval; d_RM_ = Cohen’s *d*_Repeated Measures_, effect-size measure for post hoc comparisons within groups, pairwise comparisons are Bonferroni-corrected (*p* < .05, two-tailed); significant results are marked in bold; *p < .05

**Table 3:** Pairwise comparisons of measurement time points within the four groups (T1 to T5; part 2)

	Group	T2 vs. T4		T2 vs. T5		T3 vs. T4		T3 vs. T5		T4 vs. T5	
		md [95%*CI*]	d_RM_	md [95%*CI*]	d_RM_	md [95%*CI*]	d_RM_	md [95%*CI*]	d_RM_	md [95%*CI*]	d_RM_
Daily smartphone use time	EG 1					−20.01 [−45.52, 5.50]		−1.90 [−28.78, 24.98]		18.11* [0.33, 35.89]	**.22**
	EG 2					9.38 [−16.97, 35.72]		2.00 [−25.76, 29.76]		−7.38 [−25.74, 10.99]	
	EG 3					−15.78 [−40.92, 9.33]		−23.92 [−50.39, 2.55]		−8.12 [−25.63, 9.39]	
	CG					−5.10 [−31.12, 20.93]		−10.68 [−38.10, 16.75]		−5.58 [−23.72, 12.56]	
Weekly physical activity	EG 1					21.13 [−41.90, 84.15]		13.20 [−54.53, 80.92]		−7.93 [−78.08, 62.22]	
	EG 2					102.38* [37.29, 167.48]	**.43**	76.07* [6.12, 146.02]	**.30**	−26.32 [−98.77, 46.14]	
	EG 3					121.63* [59.56, 183.69]	**.55**	110.74* [44.05, 177.44]	**.54**	−10.89 [−79.97, 58.20]	
	CG					9.01 [−55.29, 73.30]		1.90 [−67.19, 70.99]		−7.11 [−78.67, 64.46]	
PSU	EG 1	1.51* [0.60, 2.41]	**.36**	2.58* [1.51, 3.65]	**.50**	1.11* [0.31, 1.90]	**.32**	2.18* [1.21, 3.15]	**.48**	1.07* [0.27, 1.87]	**.29**
	EG 2	0.07 [−0.87, 1.00]		−0.23 [−1.34, 0.87]		−0.32 [−1.14, 0.50]		−0.62 [−1.62, 0.39]		−0.30 [−1.13, 0.53]	
	EG 3	0.49 [−0.40, 1.38]		0.28 [−0.77, 1.33]		0.17 [−0.61, 0.96]		−0.04 [−0.99, 0.92]		−0.21 [−1.00, 0.58]	
	CG	−0.30 [−1.22, 0.62]		−0.42 [−1.51, 0.67]		−0.22 [−1.03, 0.59]		−0.34 [−1.33, 0.65]		−0.12 [−0.94, 0.70]	
PMH	EG 1	−0.44 [−1.29, 0.42]		−0.80 [−1.74, 0.15]		−0.41 [−1.19, 0.36]		−0.77 [−1.76, 0.21]		−0.36 [−1.29, 0.57]	
	EG 2	0.18 [−0.70, 1.07]		0.31 [−0.67, 1.29]		0.42 [−0.38, 1.22]		0.54 [−0.48, 1.56]		0.13 [−0.83, 1.08]	
	EG 3	−0.65 [−1.49, 0.19]		−0.85 [−1.78, 0.08]		−0.21 [−0.97, 0.55]		−0.41 [−1.38, 0.56]		−0.20 [−1.11, 0.72]	
	CG	−0.03 [−0.90, 0.84]		0.07 [−0.89, 1.04]		−0.18 [−0.97, 0.61]		−0.07 [−1.08, 0.93]		0.11 [−0.84, 1.05]	
Depressive symptoms	EG 1	0.30 [−0.50, 1.10]		0.71 [−0.12, 1.54]		0.09 [−0.64, 0.83]		0.51 [−0.32, 1.33]		0.41 [−0.40, 1.23]	
	EG 2	0.32 [−0.51, 1.14]		−0.13 [−0.99, 0.73]		−0.03 [−0.79, 0.73]		−0.48 [−1.34, 0.37]		−0.45 [−1.29, 0.39]	
	EG 3	0.24 [−0.54, 1.03]		0.42 [−0.40, 1.24]		−0.01 [−0.73, 0.72]		0.17 [−0.65, 0.98]		0.17 [−0.63, 0.97]	
	CG	−0.03 [−0.85, 0.78]		−0.26 [−1.11, 0.59]		0.02 [−0.73, 0.77]		−0.20 [−1.04, 0.64]		−0.23 [−1.06, 0.60]	
Anxiety symptoms	EG 1	0.38 [−0.22, 0.99]		0.52 [−0.17, 1.21]		0.26 [−0.31, 0.83]		0.40 [−0.29, 1.09]		0.14 [−0.50, 0.78]	
	EG 2	−0.07 [−0.69, 0.56]		−0.33 [−1.04, 0.39]		−0.16 [−0.75, 0.43]		−0.42 [−1.13, 0.30]		−0.26 [−0.92, 0.40]	
	EG 3	0.11 [−0.48, 0.71]		−0.20 [−0.88, 0.48]		−0.42 [−0.98, 0.14]		−0.73* [−1.41, −0.05]	**.28**	−0.31 [−0.94, 0.32]	
	CG	−0.09 [−0.71, 0.53]		−0.37 [−1.08, 0.33]		−0.02 [−0.60, 0.56]		−0.30 [−1.00, 0.40]		−0.29 [−0.94, 0.37]	

EG = experimental group, CG = control group; PSU = problematic smartphone use, PMH = positive mental health; EG1 (smartphone reduction): *n* = 128, EG2 (physical activity increase): *n* = 120, EG3 (smartphone reduction and physical activity increase): *n* = 132, CG: *n* = 123; T1 to T5 = measurement time points; md = mean difference, *CI* = confidence interval; d_RM_ = Cohen’s *d*_Repeated Measures_, effect-size measure for post hoc comparisons within groups, pairwise comparisons are Bonferroni-corrected (*p* < .05, two-tailed); significant results are marked in bold; *p < .05

**Table 4:** Pairwise comparisons between the four groups regarding smartphone use time, physical activity, and PSU (T1 to T5)

		T1		T2		T3		T4		T5	
		md [95%*CI*]	d	md [95%*CI*]	d	md [95%*CI*]	d	md [95%*CI*]	d	md [95%*CI*]	d
Daily smartphone use time	EG 1 vs. EG2	12.35 [−22.27, 46.96]				−40.84 [−83.53, 1.84]		−11.46 [−45.52, 22.60]		−36.94* [−69.54, −4.35]	**.38**
	EG 1 vs. EG3	0.14 [−33.65, 33.94]				−11.36 [−53.03, 30.31]		−7.15 [−40.40, 26.11]		−33.38* [−65.20, −1.56]	**.35**
	EG 1 vs. CG	13.25 [−21.14, 47.65]				−34.76 [−77.17, 7.66]		−19.85 [−53.69, 14.00]		−43.53* [−75.92, −11.15]	**.56**
	EG2 vs. EG3	−12.21 [−46.56, 22.15]				29.48 [−12.89, 71.85]		4.32 [−29.50, 38.12]		3.57 [−28.79, 35.92]	
	EG2 vs. CG	0.90 [−34.05, 35.86]				6.09 [−37.02, 49.19]		−8.39 [−42.78, 26.01]		−6.59 [−39.50, 26.32]	
	EG3 vs. CG	13.11 [−21.03, 47.25]				−23.40 [−65.50, 18.70]		−12.70 [−46.30, 20.90]		−10.16 [−42.30, 21.99]	
Weekly physical activity	EG 1 vs. EG2	29.95 [−34.42, 88.32]				−97.14* [−176.50, −17.79]	**.36**	−15.88 [−106.56, 74.80]		−34.27 [−123.14, 54.60]	
	EG 1 vs. EG3	34.12 [−25.79, 94.03]				−85.33* [−162.80, −7.86]	**.34**	15.17 [−73.36, 103.70]		12.21 [−74.55, 98.98]	
	EG 1 vs. CG	−7.43 [−68.41, 53.55]				53.46 [−25.40, 132.31]		41.34 [−48.77, 131.45]		42.17 [−46.15, 130.48]	
	EG2 vs. EG3	7.17 [−53.75, 68.09]				11.81 [−66.96, 90.58]		31.05 [−58.96, 121.07]		46.48 [−41.74, 134.70]	
	EG2 vs. CG	−34.38 [−96.35, 27.59]				150.60* [70.47, 230.73]	**.70**	57.22 [−34.34, 148.79]		76.44 [−13.31, 166.18]	
	EG3 vs. CG	−41.55 [−102.07, 18.98]				138.79* [60.53, 217.06]	**.71**	26.17 [−63.26, 115.61]		29.95 [−57.70, 117.61]	
PSU	EG 1 vs. EG2	1.23 [−0.41, 2.87]		1.80* [0.20, 3.40]	**.38**	1.79* [0.11, 3.46]	**.37**	0.36 [−1.26, 1.98]		−1.01 [−2.70, 0.68]	
	EG 1 vs. EG3	−0.07 [−1.67, 1.53]		0.96 [−0.61, 2.52]		0.88 [−0.76, 2.51]		−0.06 [−1.64, 1.52]		−1.34 [−2.99, 0.31]	
	EG 1 vs. CG	0.79 [−0.84, 2.42]		1.28 [−0.32, 2.87]		0.80 [−0.87, 2.46]		−0.53 [−2.14, 1.07]		−1.73* [−3.41, −0.05]	**.35**
	EG2 vs. EG3	−1.30 [−2.93, 0.33]		−0.85 [−2.44, 0.74]		−0.91 [−2.58, 0.75]		−0.42 [−2.03, 1.19]		−0.33 [−2.01, 1.35]	
	EG2 vs. CG	−0.44 [−2.10, 1.22]		−0.53 [−2.14, 1.09]		−0.99 [−2.69, 0.70]		−0.90 [−2.53, 0.74]		−0.72 [−2.43, 0.99]	
	EG3 vs. CG	0.86 [−0.76, 2.48]		0.318 [−1.26, 1.90]		−0.08 [−1.74, 1.57]		−0.48 [−2.07, 1.12]		−0.39 [−2.05, 1.28]	

EG = experimental group, CG = control group; PSU = problematic smartphone use; EG1 (smartphone reduction): *n* = 128, EG2 (physical activity increase): *n* = 120, EG3 (smartphone reduction and physical activity increase): *n* = 132, CG: *n* = 123; T1 to T5 = measurement time points; md = mean difference, *CI* = confidence interval; d = Cohen’s *d*, effect-size measure for post hoc comparisons between groups, pairwise comparisons are Bonferroni-corrected (*p* < .05, two-tailed); significant results are marked in bold; *p < .05

**Table 5:** Pairwise comparisons between the four groups regarding positive mental health, depressive symptoms, anxiety symptoms (T1 to T5)

		T1		T2		T3		T4		T5	
		md [95%*CI*]	d	md [95%*CI*]	d	md [95%*CI*]	d	md [95%*CI*]	d	md [95%*CI*]	d
PMH	EG 1 vs. EG2	0.01 [−1.65, 1.68]		0.18 [−1.51, 1.86]		−0.03 [−1.81, 1.74]		0.80 [−0.92, 2.51]		1.28 [−0.43, 2.99]	
	EG 1 vs. EG3	0.44 [−1.18, 2.07]		0.63 [−1.02, 2.28]		0.22 [−1.52, 1.95]		0.42 [−1.26, 2.09]		0.58 [−1.09, 2.25]	
	EG 1 vs. CG	0.38 [−1.27, 2.03]		1.04 [−0.64, 2.72]		1.21 [−0.55, 2.98]		1.45 [−0.26, 3.15]		1.91* [0.21, 3.61]	**.39**
	EG2 vs. EG3	0.43 [−1.22, 2.08]		0.46 [−1.22, 2.13]		0.25 [−1.52, 2.01]		−0.38 [−2.08, 1.32]		−0.70 [−2.40, 0.99]	
	EG2 vs. CG	0.37 [−1.31, 2.05]		0.86 [−0.84, 2.57]		1.24 [−0.55, 3.04]		0.65 [−1.09, 2.38]		0.63 [−1.10, 2.35]	
	EG3 vs. CG	−0.06 [−1.70, 1.58]		0.41 [−1.25, 2.07]		1.00 [−0.76, 2.75]		1.03 [−0.66, 2.72]		1.33 [−0.35, 3.02]	
Depressive symptoms	EG 1 vs. EG2	−0.07 [−1.44, 1.31]		−0.11 [−1.42, 1.21]		0.04 [−1.28, 1.36]		−0.09 [−1.44, 1.27]		−0.95 [−2.24, 0.34]	
	EG 1 vs. EG3	−0.39 [−1.73, 0.95]		−0.12 [−1.41, 1.16]		−0.08 [−1.36, 1.21]		−0.18 [−1.50, 1.14]		−0.42 [−1.68, 0.85]	
	EG 1 vs. CG	−0.04 [−1.40, 1.32]		−0.51 [−1.81, 0.80]		−0.77 [−2.08, 0.55]		−0.84 [−2.18, 0.51]		−1.48* [−2.76, −0.19]	**.38**
	EG2 vs. EG3	−0.32 [−1.68, 1.04]		−0.02 [−1.32, 1.29]		−0.12 [−1.43, 1.20]		−0.09 [−1.43, 1.25]		0.53 [−0.75, 1.82]	
	EG2 vs. CG	0.03 [−1.36, 1.41]		−0.40 [−1.73, 0.93]		−0.81 [−2.14, 0.53]		−0.75 [−2.12, 0.62]		−0.53 [−1.83, 0.78]	
	EG3 vs. CG	0.35 [−1.01, 1.70]		−0.39 [−1.68, 0.91]		−0.69 [−1.99, 0.61]		−0.66 [−1.99, 0.67]		−1.06 [−2.34, 0.21]	
Anxiety symptoms	EG 1 vs. EG2	0.04 [−1.05, 1.13]		0.47 [−0.54, 1.48]		0.44 [−0.63, 1.50]		0.02 [−1.01, 1.06]		−0.38 [−1.45, 0.70]	
	EG 1 vs. EG3	−0.38 [−1.44, 0.69]		−0.00 [−0.99, 0.98]		0.40 [−0.64, 1.44]		−0.27 [−1.28, 0.74]		−0.72 [−1.77, 0.33]	
	EG 1 vs. CG	−0.12 [−1.21, 0.96]		−0.42 [−1.42, 0.59]		−0.61 [−1.67, 0.45]		−0.89 [−1.92, 0.14]		−1.31* [−2.38, −0.24]	**.38**
	EG2 vs. EG3	−0.42 [−1.50, 0.66]		−0.47 [−1.47, 0.53]		−0.04 [−1.09, 1.02]		−0.29 [−1.32, 0.73]		−0.35 [−1.42, 0.72]	
	EG2 vs. CG	−0.17 [−1.27, 0.93]		−0.89 [−1.91, 0.13]		−1.05 [−2.13, 0.02]		−0.91 [−1.96, 0.14]		−0.94 [−2.02, 0.15]	
	EG3 vs. CG	0.25 [−0.82, 1.32]		−0.41 [−1.41, 0.58]		−1.02 [−2.07, 0.03]		−0.62 [−1.64, 0.40]		−0.59 [−1.65, 0.47]	

EG = experimental group, CG = control group; PMH = positive mental health; EG1 (smartphone reduction): *n* = 128, EG2 (physical activity increase): *n* = 120, EG3 (smartphone reduction and physical activity increase): *n* = 132, CG: *n* = 123; T1 to T5 = measurement time points; md = mean difference, *CI* = confidence interval; d = Cohen’s *d*, effect-size measure for post hoc comparisons between groups, pairwise comparisons are Bonferroni-corrected (*p* < .05, two-tailed); significant results are marked in bold; *p < .05

Results of the ANOVA for daily smartphone use time revealed a significant main effect for measurement time point (*F*(2.59, 1290.45) = 25.591, *p* < 0.001, η^2^_p_ = 0.049), a nonsignificant main effect for group condition (*F*(3, 499) = 1.464, *p* = 0.224, η^2^_p_ = 0.009), and a significant interaction effect (*F*(7.76, 1290.45) = 4.301, *p* < 0.001, η^2^_p_ = 0.025). Pairwise comparisons within groups indicated a significantly lower smartphone use at T3, T4, and T5 compared with baseline (T1) in EG1 and EG3. These effects were stronger in EG1 than in EG3. Further, there was a significant decrease from T4 to T5 in EG1 (small effect). Pairwise comparisons between groups revealed a significantly lower smartphone use at T5 between EG1 and all the other groups (EG1 < EG3 < EG2 < CG). While the effects of the differences between EG1 and the two other experimental groups were small, the effect of the difference between EG1 and CG was moderate.

For weekly physical activity, the ANOVA displayed a significant main effect for measurement time point (*F*(2.83, 1412.33) = 26.674, *p* < 0.001, η^2^_p_ = 0.051), a significant main effect for group condition (*F*(3, 499) = 2.651, *p* = 0.048, η^2^_p_ = 0.016), and a significant interaction effect (*F*(8.49, 1412.33) = 5.127, *p* < 0.001, η^2^_p_ = 0.030). In all three experimental groups, there was a significant increase in physical activity from baseline to post-intervention measurement (T3), with a small effect in EG1 and large effects in EG2 and EG3. After the initial increase, there were significant decreases from T3 to the follow-up measurements T4 and T5 in EG2 and EG3, with small (EG2) and moderate (EG3) effects. However, the level of physical activity was higher at all measurement time points in comparison with baseline in EG2 and higher at post-intervention and follow-up 2 than at baseline in EG3. Pairwise comparisons between groups indicated higher physical activity levels at post-intervention measurement in EG2 and EG3 in comparison with EG1 (small effect sizes) and CG (medium effect sizes).

Concerning PSU, results of the ANOVA displayed a significant main effect for measurement time point (*F*(3.43, 1710.29) = 14.195, *p* < 0.001, η^2^_p_ = 0.028), a nonsignificant main effect for group condition (*F*(3, 499) = 0.990, *p* = 0.397, η^2^_p_ = 0.006), and a significant interaction effect (*F*(10.28, 1710.29) = 6.788, *p* < 0.001, η^2^_p_ = 0.039). While the level of PSU in EG1 did not change significantly from T1 to T3, there were significant decreases from T3 to T4 and T4 to T5 (small effects). The effect size of the differences between PSU at follow-up 1 in comparison with its levels at baseline and intermediate measurement (T2) was small, that of the comparisons with follow-up 2 was medium. In EG2, the level of PSU was lower at post-intervention than at baseline (small effect). In EG3, it was lower at all measurement time points in comparison with baseline (small effects). According to pairwise comparisons, the level of PSU at T2 and T3 was higher in EG1 than in EG2 (small effects). However, it was lower in comparison with CG at follow-up 2 (small effect).

Considering PMH, the ANOVA revealed a significant main effect for measurement time point (*F*(3.49, 1739.86) = 4.520, *p* = 0.002, η^2^_p_ = 0.009), a nonsignificant main effect for group condition (*F*(3, 499) = 1.472, *p* = 0.221, η^2^_p_ = 0.009), and a significant interaction effect (*F*(10.46, 1739.86) = 2.295, *p* = 0.010, η^2^_p_ = 0.014). A similar result pattern was found in EG1 and EG3: while the level of PMH did not differ between baseline and T2 and T3, respectively, it was higher at follow-up 1 and 2 in comparison with baseline (small effect sizes). The only difference between the groups was found at follow-up 2, with higher PMH values in the EG1 than in the CG (small effect).

Regarding depressive symptoms, the ANOVA pointed to a significant main effect for measurement time point (*F*(3.70, 1847.02) = 7.662, *p* < 0.001, η^2^_p_ = 0.015), a nonsignificant main effect for group condition (*F*(3, 499) = 1.010, *p* = 0.388, η^2^_p_ = 0.006), and a significant interaction effect (*F*(11.10, 1847.02) = 1.927, *p* = 0.032, η^2^_p_ = 0.011). While the only difference between measurement time points was between T1 and T5 in EG1 (with a lower level at T5), the level of depressive symptoms was lower at post-intervention in comparison with baseline in EG2. Both effect sizes were small. In EG3, depressive symptoms were lower at all measurement time points compared with baseline (small effect sizes). Considering group differences, again, there was only a difference between EG1 and CG at follow-up 2, with a lower level of depressive symptoms in EG1 (small effect).

Results of the ANOVA for anxiety symptoms revealed a significant main effect for measurement time point (*F*(3.65, 1822.77) = 11.865, *p* < 0.001, η^2^_p_ = 0.023), a nonsignificant main effect for group condition (*F*(3, 499) = 2.095, *p* = 0.100, η^2^_p_ = 0.012), and a significant interaction effect (*F*(10.96, 1822.77) = 2.696, *p* = 0.002, η^2^_p_ = 0.016). In EG1, the level of anxiety symptoms was significantly lower at follow-up 1 and 2 compared with baseline (small effects). In EG 2 and EG3, it was significantly lower at T2, T3, and T4 in comparison with baseline, with small to medium effect sizes. After this decrease, there was a slight increase (small effect) from post-intervention to follow-up 2 in EG3. Pairwise comparisons between groups indicated a significantly lower level of anxiety symptoms in EG1 than in CG at T5 (small effect).

## Discussion

Over the past few years, there has been a COVID-19 pandemic-related increase in the use of digital technology in general (Pandya and Lodha 2021; Wagner et al. 2021) and of smartphone use in particular (Jiang et al. 2022; Tyagi et al. 2021). Reasons for this development could be boredom and the desire to stay in contact with family and friends during anti-COVID-19 social-distancing measures (Garfin 2020; Wagner et al. 2021). However, even before the pandemic, there was a significant increase in smartphone screen time, especially in adolescents and young adults (Harvey et al. 2022; Twenge et al. 2018). When people rely on their smartphone to cope with stress and negative emotions or to compensate for unmet psychological needs, this can result in PSU (Brand et al. 2016; Schneider et al. 2022). PSU has increased over the past decade (Olson et al. 2020). Frequent smartphone use and PSU are linked to numerous detrimental mental health outcomes such as symptoms of depression, anxiety, and stress (Thomée 2018; Ting and Chen 2020). However, most studies that explored the links between smartphone use and mental health used cross-sectional designs. Thus, true conclusions concerning risk factors and outcomes, or rather causal directions of associations, often remained unclear (Thomée 2018). In addition, there is a need to investigate measures that are most successful in avoiding and correcting PSU to healthy levels (Busch and McCarthy 2021; Radtke et al. 2022).

Against this background, the present study employed a longitudinal experimental design to draw causal conclusions regarding the effects of simple measures for the prevention of PSU and detrimental health outcomes, and for the promotion of psychological well-being. We compared the effects of three experimental conditions: reduced smartphone use time, increased physical activity time, and a combination of both conditions. The analyses displayed significant main effects for measurement time point and significant interaction effects for all considered outcomes. The reduction of smartphone use time and the combination of both conditions led to a significant reduction in participants’ daily smartphone use and to higher levels of PMH. Furthermore, all three interventions resulted in a significant increase in weekly physical activity as well as in reduced symptoms of PSU, depression, and anxiety.

In line with Brailovskaia et al. (2022), the controlled reduction of smartphone use time over 14 days led to a significant reduction in smartphone use not only in the short but also in the longer term (confirmation of Hypothesis 1a). The intervention increased the participants’ awareness of their smartphone use, which was accompanied by a more conscious and therefore reduced usage. Furthermore, participants noted the positive effects of the reduction, which explained why the smartphone use remained at a lower level (compared with baseline) even after the intervention. In addition, the experience of being able to reduce one’s smartphone consumption might foster their perceived self-efficacy, which is an important factor in closing the intention–behavior gap (Schwarzer 2008). Contrary to our assumption, the enhancement of physical activity did not significantly reduce smartphone use time (rejection of Hypothesis 1d). It seems that the two health behaviors are not as strongly interrelated as others, such as physical activity and a healthy diet (Fleig et al. 2014; Miao et al. 2017) or smoking and alcohol consumption (Keller et al. 2008; Laaksonen et al. 2002).

However, there was a short-term effect of the reduction of smartphone use time on physical activity (partial confirmation of Hypothesis 1c). Thus, the participants could use the newly available time to engage in physical activity. This is in line with the displacement hypothesis, which posits that time spent on digital devices takes time away from other activities and health behaviors like physical activity or supportive social interactions (Przybylski and Weinstein 2017; Thomée 2018). Yet, it remains an open question why the level of physical activity of EG1 only increased in the short-term even though the reduced level of smartphone use continued in the longer term. In EG2 and EG3, where the level of physical activity was increased over the 14-day period, the level of physical activity was higher at all measurement time points compared with baseline (confirmation of Hypothesis 1b). This is in line with Fleig et al. (2014), who reported the predictive value of physical activity for long-term exercise habits. Again, it could be that the intervention increased the participants’ awareness of their physical activity levels and effort for a healthier lifestyle (Brailovskaia et al. 2022). In addition, the successful increase in physical activity during the experimental manipulation likewise could strengthen the perceived self-efficacy and thus reduce the intention–behavior gap (Schwarzer 2008). Notably, after the initial increase (short-term effect), the level of physical activity decreased again in the longer term (although it was still higher than at baseline). As it takes approximately 66 days for people to form a new habit (Lally et al. 2010), it could be useful to regularly remind people of (the benefits of) increased physical activity levels to maintain them over the long term. Notably, the present results indicate that this automation might be more rapid in the case of smartphone reduction.

Considering PSU, there were short-term effects of the increased physical activity (partial confirmation of Hypothesis 2b). The engagement in physical activity can fulfill different psychological needs (Wiese et al. 2018). For example, reaching self-imposed goals (e.g., running a certain distance) could lead to the experience of competence, while the engagement in sportive activities with others could fulfill the need for relatedness. Thus, people do not have to rely on their smartphone for need satisfaction in the online world (Cheng and Lau 2022; Schneider et al. 2022). The lack of longer term effects might be explained by the return to decreased physical activity levels after the initial increase. The effects of the smartphone reduction on PSU occurred first in the longer term (partial confirmation of Hypothesis 2a). From baseline to post-intervention measurement, the level of PSU remained constant. This corresponds to previous experimental studies that reported higher levels of symptoms of anxiety (Cheever et al. 2014; Hartanto and Yang 2016), craving (Wilcockson et al. 2019), and withdrawal (Eide et al. 2018; Sapacz et al. 2016) during or after being separated from the smartphone, especially in individuals with high PSU tendencies. Brailovskaia et al. (2022) indicated that these symptoms result not only from complete smartphone abstinence but also as a consequence of a daily reduction in use by 1 hour, which is likewise reflected in the present results. After an initial craving stage, the negative symptoms decreased (Brailovskaia et al. 2022). Similarly, we found positive effects of the smartphone reduction on PSU in the longer term. Remarkably, the combination of smartphone reduction with physical activity increase led to reduced PSU levels in both the short and longer term. Thus, the combination seems to be most effective when addressing PSU. It could be that the effects of physical activity on reward stimulation buffer the negative symptoms within the initial phase of craving which typically occur during a smartphone reduction alone.

Considering positive and negative aspects of mental health, the effects of the smartphone use reduction were found in the longer term (partial confirmation of Hypotheses 3a, 3c and 3e). This might be explained by the negative symptoms within the craving stage (e.g., Cheever et al. 2014; Eide et al. 2018) that decline over time. The increase in physical activity resulted in lower levels of depressive symptoms in the short-term (partial confirmation of Hypothesis 3d) and lower levels of anxiety symptoms in the short and partly in the longer term (i.e., immediately after and 1 month after the intervention; partial confirmation of Hypothesis 3f). The return to decreased physical activity levels after the post-intervention measurement could be the reason for the lack of a longer term effect. Again, the results underline the importance of regular physical activity over longer periods for beneficial effects on negative aspects of mental health (Rebar et al. 2015). In contrast to previous research that reported longitudinal associations or positive effects of physical activity on PMH (Doré et al. 2018; Precht et al. 2022), happiness (Zhang and Chen 2019), and well-being (Harris 2018), the present study found no effects of the physical activity intervention on PMH (rejection of Hypothesis 3b). It is probably not only the duration of the intervention but also the type of physical activity that is decisive for favorable effects: in physical activity interventions, participants usually benefit from experiencing social interactions, social support, and feelings of safety (Mason and Holt 2012). Concerning leisure-time physical activity, specifically the context of informal group activities and team sports provides the beneficial effects on PMH (Doré et al. 2018; Eime et al. 2013). As COVID-19-related social distancing measures were partially present during the investigation period (Bundesministerium für Gesundheit 2022), the participants of the present study possibly carried out their physical activity alone. Thus, they could not profit from social cohesion or team spirit that could accompany activities in groups. The effects of the combination of both interventions on PMH were similar to the effects of the smartphone reduction alone. The effects on anxiety symptoms were comparable to the effects of the physical activity increase alone. Concerning depressive symptoms, the combination produced positive effects at all measurement time points.

To summarize, all three interventions had a positive effect on the investigated variables. However, they differed in their effectiveness (see *Research Question 1*). Apart from its influence on the physical activity level, the reduction in daily smartphone use time by 60 minutes was more effective than the increase in daily physical activity by 30 minutes considering the smartphone-related constructs of daily use time and PSU, PMH, and symptoms of depression and anxiety (see *Research Question 2*). Furthermore, the combination of both measures was of great benefit. It yielded positive effects similar to the reduction in smartphone use time alone. However, these positive effects partly occurred earlier than the effects of the reduction alone (see *Research Question 2*). Thus, experiencing the positive effects earlier in time might motivate persons to further engage in a healthier lifestyle. However, the effects of the reduction in smartphone use time alone were often more stable (see *Research Question 2*). Therefore, it could be beneficial to modify the increase in physical activity (also when it is combined with the smartphone reduction): instead of asking individuals to increase their daily physical activity level by 30 minutes for 2 weeks, it seems desirable to offer structured, group-based programs for several months (e.g., Eime et al. 2013; Liu et al. 2019; Mason and Holt 2012). Alternatively, against the background of easily applicable, cost-effective measures, people could be constantly motivated—at least for a period of 66 days (Lally et al. 2010)—to regularly engage in aerobic endurance exercise (Yang et al. 2021), preferably with other persons (Doré et al. 2018).

Despite the practical significance of the current results, some limitations of the present study are noteworthy. First, most of the effect sizes were small to medium. As non-compliers were kept in the sample for the analyses, the results can be considered as conservative estimates of the true treatment effects which might be larger in size (Tromholt 2016). However, even small effect sizes can have far-reaching consequences, especially when they accumulate over time (Thompson 2007). Second, the interventions were derived from theoretical considerations and average values (reduction of smartphone use) as well as general recommendations on health care (increase in physical activity). It would be desirable to tailor the interventions explicitly to each participant instead of aiming at an absolute reduction in smartphone use time as well as an increase in physical activity level. For some participants, the reduction of daily use time by 1 hour might have meant nearly complete abstinence from smartphone use, whereas others possibly only reduced their consumption from, for example, 6 to 5 hours. While researchers have already established evidence regarding the optimal dose–response for physical activity and mental health (Bernard et al. 2018; Kim et al. 2018, 2020), the “sweet spot” for smartphone use requires further examination (Brailovskaia et al. 2022). Third, the predominantly female composition of the sample probably reduced the generalizability of the results. Since female persons are more vulnerable to PSU than male persons (Busch and McCarthy 2021), future research should strive for gender-balanced samples. Fourth, a possible attrition bias must be noted. Over the investigation period of 3.5 months, dropout rates ranged from 9% to 17%. Considering demographic variables (i.e., age, gender, marital status, and occupational status), there were no differences between participants who dropped out and those who participated in all surveys. However, intention-to-treat analysis was not possible due to a lack of willingness to continue answering the surveys after dropping out of the study. Therefore, further investigation of the time point (i.e., during the 14-day intervention period or the follow-up period) and the reasons (e.g., lack of motivation to be physically active, to fill in the compliance diary, or to answer the surveys) at which people stopped participating would be desirable for the generalizability of the results as well as for adapting the interventions for better feasibility. Fifth, conclusions of the present study were based on self-reporting. Therefore, results may underlie misclassification, recall difficulty, recall bias, or response-style bias (Silfee et al. 2018; Thomée 2018). As previous research has shown that objective measures are more precise than subjective measures (Boase and Ling 2013; Prince et al. 2008), future studies should focus primarily on participants’ phone logs (Elhai et al. 2019) and data from wearable monitors (e.g., accelerometers, pedometers, and heart rate monitors; Silfee et al. 2018). Finally, it would be worthwhile to assess the use of other electronic devices apart from the smartphone (e.g., laptops or tablets), which would reveal possible compensatory behaviors of the participants (Radtke et al. 2022).

## Conclusion

In summary, over the past decade, significant increases have been noted in smartphone screen time (Harvey et al. 2022) and PSU (Olson et al. 2020), which is often associated with numerous detrimental mental health outcomes (Thomée 2018). Therefore, the present study compared the influence of three simple measures that were suggested to have a beneficial influence on PSU tendencies and positive and negative aspects of mental health. The 14-day reduction of daily smartphone use time by 60 minutes was more effective than the increase in daily physical activity by 30 minutes within the same period considering PSU and variables of mental health. The combination of both interventions revealed predominantly comparable longer term effects to reduced usage alone. In addition, the combination seemed to reduce the negative symptoms within the initial phase of craving which typically occur during smartphone reduction alone. Thus, the results indicate an interrelation between the health behaviors of smartphone use and physical activity. If they are combined, they can be applied as an efficient, low-threshold, and cost-effective measure to prevent PSU and its detrimental mental health outcomes and thus foster psychological well-being.

## Data Availability

The dataset generated and analyzed during the current study is available from the corresponding author upon reasonable request.
